# Supplementary material for: Infection prevention and control risk factors in health workers infected with SARS-CoV-2 in Jordan: A case control study
Source: PLoS One. 2022 Jul 8;17(7):e0271133. doi: 10.1371/journal.pone.0271133 (PMC9269456; doi:10.1371/journal.pone.0271133)
Supplement: Attachment — Submitted filename: rebuttal letter.docx [file pone.0271133.s001.docx]

Reviewer #1: Thanks for the authors for the good work, comments as below:

1- In introduction section line -75 & discussion section line-318, It would be great if the COVID-19 epidemiological curve in Jordan during the first 19 months of the pandemic can be demonstrated in a figure that reflect count of confirmed covid-19 cases in community and counts of infected HWs in 8 hospitals in parallel with actions taken at the public & hospital level. See an example below:
<https://doi.org/10.1001/jama.2020.11160>

- We acknowledge the important contribution that the suggested graph would add’ however, we have no comprehensive time-data around infections in healthcare workers

2- Please indicate what is the status of 8 hospitals in regard to similarities in IPC practices, PPE shortages and designated COVID-19 hospitals.

- Thanks for the important input and we added as follows: “*At the time of the study, all selected facilities were designated as COVID-19 hospitals, with adequate stock in personal protective equipment, and IPC practices were aligned under the standard protocol devised the Ministry of Health of Jordan with the support of the WHO Jordan Country Office. Specifically, training on the use of personal protective equipment (PPE), including hand washing steps and its complementarity with the use of gloves (remove gloves and proceed to hand washing between patients or between contact with various sites on a single patient), had been ongoing in line with international standards and with practical sessions.”*

3- Under study design & participants enrollment:
Line -115, please define a suspected/probable COVID-19 patients or indicate the references in reference section.

- As suggested, we indicated the appropriate reference

4- Under identification of cases subsection: RDT abbreviation stands for what- need to write it as is in the first time.? Also, what do you mean by suspected criteria A or B in line 124 (please clarify).

- We agree on the importance of spelling out and clarified RDT being rapid diagnostic test. We also added the previous reference to clarify that we are referring to the WHO probable and suspected criteria

5- Under control section: How did you manage HWs with prior positive PCR or RDT , did you test them before enrollment especially they can carry the virus with no symptoms, how can you handle it.

- We better clarified this aspect and highlighted the fact that controls were tested before enrollment. The sentence now reads: *“Enrolment was preceded by testing and having a positive serology test to SARS-CoV-2 and/or prior vaccination was considered as exclusion criteria”*

Reviewer #2: This study supports the COVID-19 prevention measures adopted in hospitals located in Jordan as advised by the WHO. The results highlights the importance of personal hygiene in combating viral infections as well as in precautions to pandemic. The study is short but has impact in the current situation.

Minor comments
1. The standard method of hand washing needs to be mentioned with other PPEs. A short summary of the training provided to HWs needs to be mentioned.

- We clarified and added as follows: “*At the time of the study, all selected facilities were designated as COVID-19 hospitals, with adequate stock in personal protective equipment, and IPC practices were aligned under the standard protocol devised the Ministry of Health of Jordan with the support of the WHO Jordan Country Office. Specifically, training on the use of personal protective equipment (PPE), including hand washing steps and its complementarity with the use of gloves (remove gloves and proceed to hand washing between patients or between contact with various sites on a single patient), had been ongoing in line with international standards and with practical sessions.”*

2. Whether the HWs were trained for hand washing steps, especially for this study?

- Please see above

3. The HWs were wearing gloves as part of their PPEs. How does the investigators implement hand washing in this context? Do they change the gloves very often during their work?

- Please see above

4. Authors stated that HWs exposed to COVID-19 patients within 1 meter for more than 15 minutes increased 3-fold adds of infection. As SARS-CoV-2 is a respiratory virus, a more detailed clarification with respect to the face mask need to be described.

We expanded on this concept and added: “*While our findings on physical distancing of at least 1 metre confirms it as a key IPC and public health and social measure to reduce transmission of SARS-CoV-2, the available evidence mostly stems from studies with small sample sizes and other methodological concerns (22,23,24). On the other hand, this confirms the importance of physical distancing as part of a comprehensive strategy of measures to suppress transmission, including mask wearing (use of masks alone not sufficient to provide an adequate level of protection against COVID-19).”*
