# Supplementary material for: Infection prevention and control risk factors in health workers infected with SARS-CoV-2 in Jordan: A case control study
Source: PLoS One. 2022 Jul 8;17(7):e0271133. doi: 10.1371/journal.pone.0271133 (PMC9269456; doi:10.1371/journal.pone.0271133)
Supplement: Attachment — Submitted filename: response to reviewers.docx [file pone.0271133.s002.docx]

Editor comments:

*As stated in the previous communication, please:*

1) *clarify the study period and*
2) *any information on VOC/VOIs on the impact of your findings.*

Dear Editor, thanks for your inputs and please find below responses to your queries:

1) We added in the abstract the following statement (lines 40-41): The study lasted approximately two months (from early January to early March 2021).

A very similar statement was added in the Introduction (lines 97-98)

2) We added the following statement in the Discussion (lines 333-336): Finally, generalization is not possible due to important differences in the pandemic evolving differently across countries, regions, and hospitals. Specifically, during the study period health workers were most likely affected by the original coronavirus strain, which was less contagious than the subsequent VOCs.
